# A chromosome-level genome assembly of *Prosopocoilus inquinatus* Westwood, 1848 (Coleoptera: Lucanidae)

**DOI:** 10.1038/s41597-024-03647-9

**Published:** 2024-07-20

**Authors:** Bo Pang, Zhihong Zhan, Yunchao Wang

**Affiliations:** 1 Plant Protection Department, College of Agriculture and Animal Husbandry of Xizang Autonomous Region, Lhasa, 850000 China; 2 Department of Entomology, College of Plant Protection, Nanjing Agricultural University, Nanjing, 210095 China; 3 College of Biology and Agriculture, Zunyi Normal University, Zunyi, 563006 China

**Keywords:** Evolutionary genetics, Genome

## Abstract

Lucanidae (Coleoptera: Scarabaeidae) are fascinating beetles exhibiting significant dimorphism and are widely used as beetle evolutionary study models. However, lacking high-quality genomes prohibits our understanding of Lucanidae. Herein, we proposed a chromosome-level genome assembly of a widespread species, *Prosopocoilus inquinatus*, combining PacBio HiFi, Illumina, and Hi-C data. The genome size reaches 649.73 Mb, having the scaffold N50 size of 59.50 Mb, and 99.6% (647.13 Mb) of the assembly successfully anchored on 12 chromosomes. The BUSCO analysis of the genome exhibits a completeness of 99.6% (n = 1,367), including 1,362 (98.5%) single-copy BUSCOs and 15 (1.1%) duplicated BUSCOs. The genome annotation identifies that the genome contains 61.41% repeat elements and 13,452 predicted protein-coding genes. This high-quality Lucanidae genome provides treasured genomic information to our knowledge of stag beetles.

## Background & Summary

The stag beetle (Coleoptera: Lucanidae) is a family in Superfamily Scarabaeoidea, comprising around 1,500 species worldwide^1^. Most stag beetle species exhibit significant intraspecific or even interspecific sexual dimorphism, in which males usually tend to have extremely impressive mandibles to fight and attract females in the wild. Thus, stag beetles have received much attention since Linnaeus first described the *Scarabaeus parallelipipedus* from Europe (later transferred to the genus *Dorcus*)^2^. Many lucanid species have been selected as an ideal behavior and functional morphology study model, and their fascinating mandibles make them popular pets and valuably private collections^3–7^. In the wild, most stag beetles are closely related to forest ecosystems, as their carboxylic larvae usually feed on decaying logs and other litter, such as leaves or fungi^8–10^.

The major geographical distribution and species diversity of Lucanidae are associated with the Indomalayan and Palearctic regions; 33 genera and nearly 400 species are known from China^11–13^. The present research on the stag beetle primarily focuses on its taxonomy and phylogeny, including new species descriptions and mitochondrial genome studies^7,11–14^. Our understanding of the stag beetle genome, especially high-quality genome assembly, remains in its infancy. Only one genome, *Dorcus hopei*, has been reported^15^. Compared with other beetles’ sharply increasing genome assembly number, more high-quality genome assemblies for stag beetles have become necessary and inevitable.

To enhance the knowledge of the taxonomy, evolution, and ecology of Lucanidae, we proposed the chromosome-level genome of a widespread species, *Prosopocoilus inquinatus* (Westwood, 1848), with the combination of PacBio HiFi, Illumina, and Hi-C data. Genome annotation, including repeats, non-coding RNAs (ncRNAs), and protein-coding genes (PCGs) were analyzed and exhibited. The high-quality genome of *P. inquinatus* provides valuable genomic information for Lucanidae study.

## Methods

### Sample collection and sequencing

A single *P. inquinatus* male sample was collected for DNA and RNA sequencing data on April 30, 2023, in Motuo County, Xizang, China. Muscle tissue, including the pronotum and posterior abdomen, was extracted from the specimen and washed via phosphate-buffered saline (PBS) solution for five minutes to eliminate any possible external pollutants. The specimen was then transferred into liquid nitrogen, frozen for at least 20 minutes, and kept at −80 °C for temporary storage until sequencing.

The specimen’s genomic DNA (gDNA) was extracted using the FastPure® Blood/Cell/Tissue/Bacteria DNA Isolation Mini Kit (Vazyme Biotech Co., Ltd, Nanjing, China). High molecular weight (HMW) gDNA was sheared into 15 kb with the MegaruptorTM device (Diagenode, Liege, Belgium) and was enriched using the AMPurePB Beads. PCR-free short reads library for whole genome sequencing (WGS) was prepared using the Truseq DNA PCR-free Kit. A PacBio HiFi 15 kb library was prepared using the SMRTbellTM Express Template Prep Kit 2.0, and the resulting library was sequenced on the PacBio Sequel II platform. The Hi-C data was carried out by digesting extracted DNA with the Mbol restriction enzyme. RNA was lysed from the specimen using the TRIzoTM Reagent (Invitrogen, Carlsbad, CA, USA). RNA-seq libraries were constructed using the VAHTS mRNA-seq v2 Library Prep Kit (Vazyme, Nanjing, China). The Illumina NovaSeq. 6000 platform was used to build all short-read libraries. The Nanopore PromethlION platform constructed long reads of the RNA library. Berry Genomics (Beijing, China) carried out all library constructions and sequencing. Consequently, we obtained 272.73 Gb of sequencing data, including 109.10 Gb (152.68×) of Illumina reads, 42.50 Gb (65.41×) of PacBio HiFi reads, 101.03 Gb (155.40×) of Hi-C data, 20.10 Gb of transcriptome data, including 9.72 Gb of short reads data and 10.38 Gb of long reads data (RNA-ONT) (Table 1).

**Table 1:** Statistics of the sequencing data generated for *Prosopocoilus inquinatus*.

Libraries	Insert sizes (bp)	Raw data (Gb)	Coverage (x)
Illumina	150	109.10	152.68
PacBio	15 Kb	42.50	65.41
Hi-C	350	101.03	155.40
RNA	350	9.72	—
RNA-ONT	5 Kb	10.38	—

### ***De novo*** genome assembly

Raw genomic Illumina sequencing reads for genome scan were employed as quality control using Fastp v0.23.2^16^ to remove adaptors, duplications, and low-quality reads.

Raw PacBio HiFi reads were generated into the primary assembly using Hifiasm v0.19.8^17^. The direct reads were then mapped with the raw HiFi reads using Minimap2 v2.24^18^ to calculate the mapping rate. One round of primary self-polishing assembly was performed for primary assembly by utilizing NextPolish2 v0.2.0^19^.

Raw Hi-C data was processed under quality control to remove duplicates using Chromap v.0.2.5-r473^20^. Clean Hi-C data was then utilized to align the primary assembly for haplotype identification and division. Contigs were anchored and orientated onto chromosomes using YaHS v1.2^21^ and Juicer v1.6.2^22^. The result of the contig assembly was reviewed, and any assembly errors were corrected manually under Juicebox v.1.11.08^23^. To determine the autosomes and sex chromosomes, the final assembly was remapped with raw HiFi data by using MiniMap2 to determine each chromosome length. Chromosome coverage was then calculated using SAMtools v. 1.9^24^ by dividing raw data by chromosome length. Moreover, the X chromosome was also detected by chromosome synteny between the model beetle species, *Tribolium castaneum*, and the relative species *Trypoxylus dichotomus* according to the relatively conserved feature in insect sexual chromosome X^25^. Syntenic blocks were identified and determined using MCScanX^26^ and TBtools^27^. Conclusively, the X chromosome was identified by exhibiting around half of the chromosome coverage compared with other chromosomes (Table 3) and re-confirmed by sharing high synteny features with other beetles’ X chromosomes (Fig. 2).

To ensure the high-quality assembly of our genome, potential contaminants were detected and eliminated by software and NCBI. In this case, we focused on Humans, Bacteria, viruses, and plant sequences. Possible contaminants were detected using MMseq. 2 v11^28^, which utilizes BLASTN-like searches and the UniVec database based on the NCBI nucleotide database. Potential vector contaminants were also specifically detected and identified by blastn (BLAST + v2.11.0^29^) against the UniVec database. Sequences with over 90% hits in the database above were considered contaminants, and sequences with over 80% hits were rechecked by online BLASTN analysis in the NCBI nucleotide database. The final genome assembly was uploaded to NCBI to detect and eliminate contaminants. According to vector search, no prominent contaminant was found in our assembly, reflecting the high quality of sample preparation and accuracy of specimen sequencing.

**Fig. 1:**
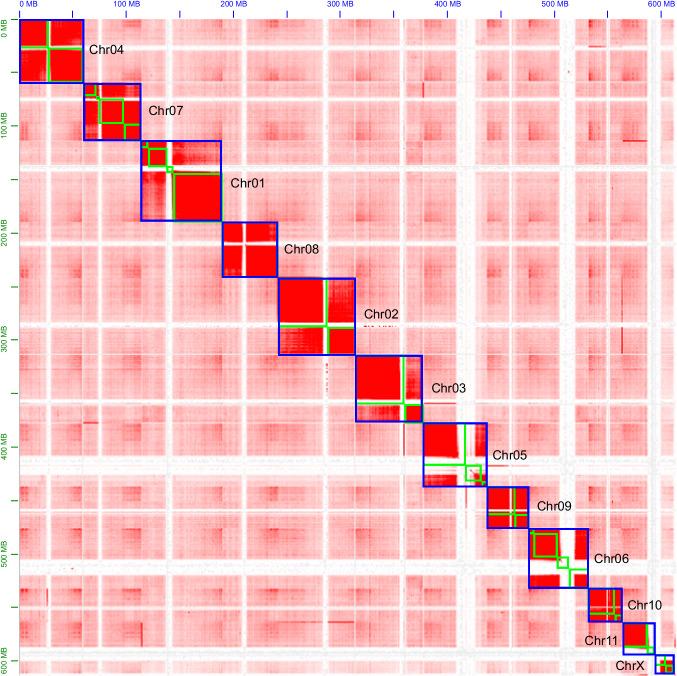
Genome-wide chromosomal heatmap of *Prosopocoilus inquinatus*, with each chromosome and contig framed in blue and green, respectively. “ChrX” represented the sex chromosome.

The final *P. inquinatus* genome assembly eventually reached the chromosomal level with a total size of 649.73 Mb, consisting of 174 scaffolds and 195 contigs (Table 2). The scaffold and contig N50 length reached 59.5 Mb and 26.36 Mb, respectively. GC content of the *P. inquinatus* was 35.67%. Most contigs (612.12 Mb, 94.21%) were firmly anchored and orientated onto 12 chromosomes. All chromosome coverage was computed and exhibited (Table 3). Among these chromosomes, one particular chromosome, number 12, has a coverage of 37.02 for long-read sequencing and 88.58 for short-read sequencing, around half of the other chromosomes (Table 3). Hence, the number 12 chromosome was considered the X chromosome in *P. inquinatus*. All chromosomes in assembly, including 11 autosomes and X chromosome, with individual lengths ranging from 17.22 to 75.68 Mb (Tables 2, 3; Fig. 1). Compared with the assembly result of its related species, *Trypoxylus dichotomus*^30^ (Sarabaeidae) (636.37 Mb in genome size and 35.11% GC content), *P. inquinatus* exhibited a larger genome size and GC content (Table 4).

**Table 2:** Genome assembly statistics for *Prosopocoilus inquinatus*.

Assembly	Total length (Mb)	Number scaffolds/contigs	Scaffold/contig N50 length (Mb)	GC (%)	BUSCO (n = 1,367) (%)			
					C	D	F	M
Hifiasm	663.65	426/426	26.74/26.74	36.07	99.7	1.5	0.1	0.2
NextPolish	650.18	195/195	26.74/26.74	35.67	99.7	1.5	0.1	0.2
Yahs	650.18	176/197	59.50/26.36	35.67	99.6	1.1	0.1	0.3
Final	649.73	174/195	59.50/26.36	35.67	99.6	1.1	0.1	0.3

C: complete BUSCOs; D: complete and duplicated BUSCOs; F: fragmented BUSCOs; M: missing BUSCOs.

**Table 3:** Chromosome status of *Prosopocoilus inquinatus*.

Chromosome Number	Length (Mb)	Coverage Long Reads	Coverage Short Reads
1	75.68	71.51	161.05
2	72.35	73.27	162.61
3	63.16	71.78	163.00
4	60.00	71.33	163.36
5	59.50	58.58	134.48
6	56.11	63.16	146.63
7	54.96	77.14	167.57
8	51.48	71.28	162.93
9	39.28	68.16	155.66
10	31.60	65.72	150.16
11	30.77	62.92	145.08
X	17.23	37.02	88.58

**Fig. 2:**
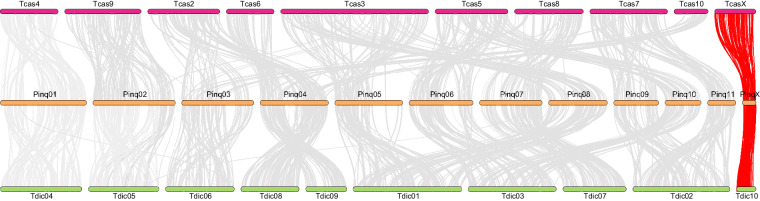
Chromosomal synteny between *Tribolium castaneum*, *Prosopocoilus inquinatus, and Trypoxylus dichotomus. The sexual chromosome X is labeled red*.

**Table 4:** Genome assembly and annotation statistics for *Prosopocoilus inquinatus* and its relative species, *Trypoxylus dichotomus* (Scarabaeidae).

Species	*P. inquinatus*	*T. dichotomus*
Genome assembly		
Size (Mb)	649.73	636.37
Number of scaffolds	174	417
Number of chromosomes	12	10
Scaffold N50 length (Mb)	59.50	71.04
GC (%)	35.67	35.11
BUSCO completeness (%)	99.6	98.7
Protein-coding genes		
Number	13,452	12,193
Mean gene length (bp)	17,402	15,150
BUSCO completeness (%)	99.6	95.8
Repetitive elements		
Size (%)	62.19	57.45
DNA transposons (%)	7.33	28.97
SINEs (%)	0	0.52
LINEs (%)	3.42	9.69
LTRs (%)	8.36	1.24
Unclassified (%)	42.02	16.67
ncRNAs		
rRNA	1,004	43
miRNA	93	57
snRNA	55	129
ribozyme	6	2
tRNA	351	361
lncRNA	4	2
Others	344	74
Total number of ncRNAs	1,857	668

### Genome annotation

**Fig. 3:**
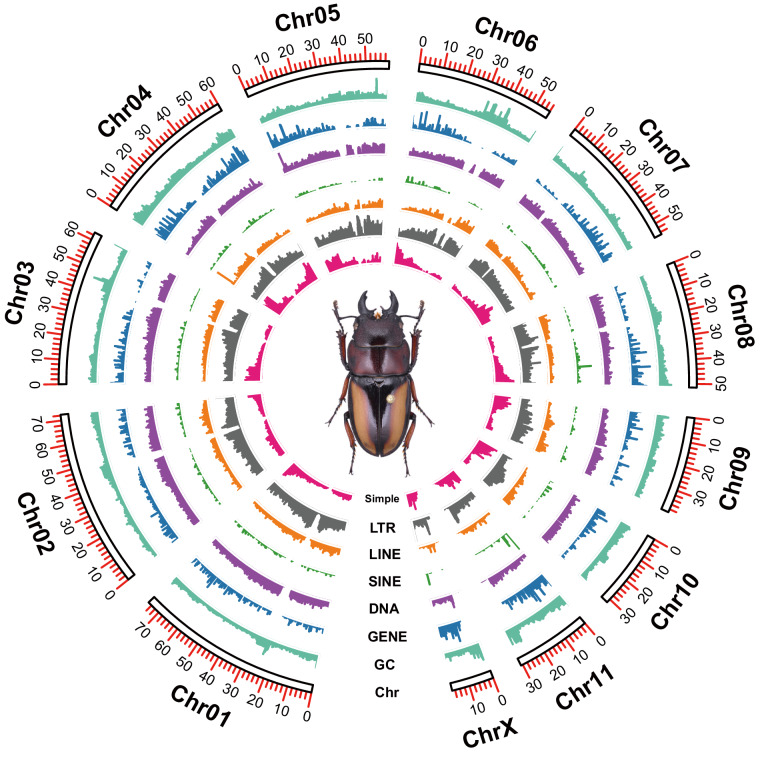
Genome characteristics of *Prosopocoilus inquinatus*. Circos plot showing the genomic characters of *P. inquinatus* from outer to inner: chromosome length (Chr) (Mb), the density of GC content (GC), the density of protein-coding genes (GENE), the density of TEs (DNA, SINE, LINE, and LTR), and simple repeats (Simple). (The sliding window size is counted for every 10 kb).

A *de novo* specific repeat library for *P. inquinatus* was built by RepeatModeler v2.0.4^31^. This specific repeat library was combined with RepBase-20230909^32^ and added to the custom library. Repeat elements in *the P. inquinatus* genome were recognized and masked by RepeatMasker v.4.1.4^33^ by aligning the custom library. Repetitive elements analysis resulting from RepeatMasker demonstrated that the *P. inquinatus* genome contains approximately 62.19% repetitive elements, including unclassified elements (42.02%), LTR elements (8.36%), DNA transposons (7.33%), LINE (1.77%), and simple repeats (0.68%) with other elements (S Table). The density for the type of each element, including simple and TEs elements, was exhibited on each chromosome (Fig. 3). Compared with the repetitive element components in *T. dichotomus*, *P. inquinatus* showed more significant size percent of Unclassified (42.02% to 16.67%) and LTR (8.36% to 1.24%) elements; however, *P. inquinatus* had a significantly minor size percent of DNA transposons, LINEs, and SINEs (Table 4).

Non-coding RNAs (ncRNAs) and transfer RNA (tRNA) in *P. inquinatus* were detected and identified by Infernal v1.1.4^34^ and tRNAscan-SE v2.0.9^35^, respectively. As a result, 1,857 ncRNAs were placed in the *P. inquinatus* genome, including four long non-coding RNAs, six ribozymes, 55 small nuclear RNAs, 93 microRNAs, 344 other ncRNAs, 351 tRNAs, and 1,004 ribosomal RNAs (Table 4). Comparatively, the number of *P. inquinatus* ncRNAs was around 2.8 times more than *T. dichotomus* (Table 4).

Protein-coding genes (PCGs) annotation in *P. inquinatus* was analyzed by MAKER v3.01.03^36^ from transcribed RNA, *ab initio* gene predictions, and homologous proteins. Transcribed RNA alignment prediction was performed by HISAT2 v2.2.1^37^. RNA-seq alignment production was then acted as a genome-guided assembly by StringTie v2.1.6^38^. The BRAKER v3.0.3^39^ was applied to acquire the *ab inito* gene predictions by employing GeneMark-ETP^40^ and Augustus v3.4.0^41^ and automatically trained them based on RNA sequence alignments and reference proteins obtained from OrthoDB v11 database^42^. GeMoMa v1.9^43^ analyzed protein-homology alignments from five insect species’ proteins, including two Coleopteran species, *Tribolium castaneum* (GCF_000002335.3^44^) and *Coccinella septempunctata* (GCF_907165205.1^45^) related to Lucanidae and three sister families of Coleoptera, including one Dipteran species, *Drosophila melanogaster* (GCA_000001215.4^46^), one Hymenopteran species, *Apis mellifera* (GCA_003254395.2^47^), and one Neuropteran species *Chrysoperla carnea* (GCA_905475395.1^48^) (Table 5). Results from BRAKER and GeMoMA were finally combined and applied as the *ab inito* input for MAKER. The final result of *P. inquinatus* PCGs establishment indicated 13,452 genes with an average length of 17,401.8 bp (Table 6).

**Table 5:** Species taxonomic information and accession code of all samples used in this study.

Species	Order	Family	Source
*Apis mellifera*	Hymenoptera	Apidae	NCBI (GCA_003254395.2)
*Chrysoperla carnea*	Neuroptera	Chrysopidae	NCBI (GCA_905475395.1)
*Coccinella septempunctata*	Coleoptera	Coccinellidae	NCBI (GCF_907165205.1)
*Drosophila melanogaster*	Diptera	Drosophilidae	NCBI (GCA_000001215.4)
*Prosopocoilus inquinatus*	Coleoptera	Lucanidae	This study
*Tribolium castaneum*	Coleoptera	Tenebrionidae	NCBI (GCF_000002335.3)

**Table 6:** Summary statistics of genome annotations in the *Prosopocoilus inquinatus* genome.

Structure annotation	
Number of protein-coding genes	13,452
Number of predicted protein sequences	17,233
Mean protein length (aa)	590.70
Mean gene length (bp)	17,401.80
Gene ratio (%)	36.03
Number of exons per gene	6.4
Mean exon length (bp)	347.40
Exon ratio (%)	4.62
Number of CDSs per gene	6.10
Mean CDS length (bp)	270.30
CDS ratio (%)	3.44
Number of introns per gene	5.30
Mean intron length (bp)	3,063
Intron ratio (%)	34.21
**Function annotation**	
Number of genes matching Uniprot records	12,719
Number of genes labeled as “Uncharacterized protein”	197
Number of genes labeled as “unknown function”	755
Number of genes with InterProScan annotations	11,503
Number of genes with GO items from InterProScan annotations	7,087
Number of genes with KEGG pathway items from InterProScan annotations	0
Number of genes with eggNOG annotations	12,434
Number of genes with GO items from eggNOG annotations	8,981
Number of genes with Enzyme Codes (EC) from eggNOG annotations	2,838
Number of genes with KEGG ko terms from eggNOG annotations	8,004
Number of genes with KEGG pathway terms from eggNOG annotations	4,924
Number of genes with COG Functional Categories from eggNOG annotations	11,656
Number of genes with GO items (combining InterProScan and eggNOG results)	10,254

The functional gene annotation was proposed by searching the UniProtKB (SwissProt and TrEMBL) 20190527 database, which uses Diamond v2.0.11.1^49^. Protein domain identifications were performed by eggNOG-mapper v2.1.9^50^ and InterProScan 5.60–92.0^51^ for Gene Ontology (GO) and KEGG pathway annotation analysis. Five databases, including Pfam^52^, SMART^53^, Superfamily^54^, Gene3D^55^, and CDD^56^, were analyzed in InterProScan. Functional annotation indicated that *P. inquinatus* contained 11,656 COG categories, 7,087 GO terms, 4,924 KEGG pathways, and 2,838 Enzyme Codes based on the InterProScan and eggNOG annotation integration (Table 6).

## Data Records

The raw sequencing data and genome assembly of *Prosopocoilus inquinatus* have been deposited at the National Center for Biotechnology Information (NCBI). The Illumina, PacBio, Hi-C, transcriptome short reads, and transcriptome long reads data can be found under identification numbers SRR27127825^57^, SRR27243604^58^, SRR27127828^59^, SRR27127827^60^, and SRR27127826^61^, respectively, under the BioProject accession number PRJNA1015594 and BioSample accession number SAMN37358649. The assembled genome has been deposited in the GeneBank in NCBI under accession number GCA_036172665.1^62^. The annotation results for repeated sequences, gene structure, and functional prediction have been deposited in the Figshare database^63^.

## Technical Validation

Berry Genomics (Beijing, China) carried out the DNA extraction. Two quantities, including the NanoDrop and Qubit, were mentioned during the extraction process (Table 7). Our extraction yielded a NanoDrop of 86 ng/μl and a 44.65 ng/μl Qubit. The 280/260 and the 260/230 of our stag beetle are 1.78 and 1.85, respectively.

**Table 7:** DNA extraction of the *Prosopocoilus inquinatus*.

NanoDrop ng/μl	260/280	260/230	Qubit ng/μl	Concentration ng/μl	Volume μl	Total amount μg
86.1	1.78	1.85	44.65	44.65	190	8.48

Two methods were used to evaluate the quality of the genome assembly. Firstly, BUSCO v5.4.4^64^ was applied for assembly completeness calculation with the reference Insecta gene set (n = 1,367) with the euk_genome_met mode. The final genome assembly showed a BUSCO completeness of 99.6%, including 1,362 (98.5%) single-copy BUSCOs, 15 (1.1%) duplicated BUSCOs, 1 (0.1%) fragmented BUSCOs, and 4 (0.3%) missing BUSCOs. To investigate the quality of the *de novo* assembly, Merqury v1.3^65^ was performed to identify possible assembly sequence errors based on efficient k-mer set operations and QV score calculation. Consequently, the k-mer completeness value of the stag beetle is 94.2%, and the QV score is 46.60. Both the k-mer value and the QV score reflect the high accuracy of the base pairs, combined with the BUSCOs, which exhibit the high completeness and accuracy of our genome assembly. The final annotation validation was also calculated by BUSCOs with a protein mode with the reference Insecta gene set (n = 1,367). The final annotation genome exhibited a BUSCO completeness of 99.6%, including 1,079 (78.9%) single-copy BUSCOs, 283 (20.7%) duplicated BUSCOs, 1 (0.1%) fragmented BUSCOs, and 4 (0.3%) missing BUSCOs. The mapping rate was also measured to determine the assembly accuracy. The mapping rates for PacBio, Illumina, RNA short reads, and RNA long reads were 99.6%, 96.51%, 96.93%, and 97.59%, respectively. These evaluations altogether reflected the high-quality value of the genome assembly.

### Supplementary information

S Table Repeat annotation in the *Prosopocoilus inquinatus* genome.

## Data Availability

All commands and pipelines used in data processing were executed according to the manual and protocols of the corresponding bioinformatic software. The settings and parameters of software were listed below: (1) Fastp v0.23.2: ‘-D’ (drop the duplicated reads), ‘-g’ (tail trimming), ‘-x’ (polymer trimming on 3′ ends), ‘-5’ (move a sliding window from 5′ tail to tail), ‘-u 10’ (unqualified percentage limit), ‘-c’ (overlapped bases correction); (2) Hifiasm v0.19.8: ‘-l2’ (strongly remove haplotig duplications); (3) Minimap2 v2.24: default parameters; (4) NextPolish2 v0.2.0: default parameters; (5) YaHS v1.2: default parameters; (6) Juicer v1.6.2: default parameters; (7) Juicebox v.1.11.08: default parameters; (8) MMseq2 v11: default parameters with ‘--search-type 3’, ‘—min-seq-id 0.8’ for potential contaminants; (9) SAMtools v. 1.9: default parameters; (10) RepeatModeler v2.0.4: ‘-LTRStruct’ LTR discovery pipeline; (11) RepeatMasker v.4.1.4: default parameters; (12) Infernal v1.1.4: default parameters; (13) tRNAscan-SE v2.0.9: ‘EukHighConfidenceFilter’ script with default parameters; (14) MAKER v3.01.03: default parameters; (15) HISAT2 v2.2.1: default parameters; (16) StringTie v2.1.6: default parameters; (17) BRAKER v3.0.3: default parameters; (18) GeneMark-ETP: default parameters; (19) Augustus v3.4.0: default parameters; (20) GeMoMa v1.9: ‘GeMoMa.m = 15000’, ‘ERE.c = false’ with default parameters; (21) Diamond v2.0.11.1: default parameters; (22) eggNOG-mapper v2.1.9: default parameters; (23) InterProScan 5.60–92.0: default parameters.
